# The Influence of Initiator Concentration on Selected Properties of Thermosensitive Poly(Acrylamide-co-2-Acrylamido-2-Methyl-1-Propanesulfonic Acid) Microparticles

**DOI:** 10.3390/polym13070996

**Published:** 2021-03-24

**Authors:** Agnieszka Gola, Andrea Bernardi, Gianfranco Pasut, Witold Musiał

**Affiliations:** 1 Department of Physical Chemistry and Biophysics, Pharmaceutical Faculty, Wroclaw Medical University, Borowska 211, 50-556 Wroclaw, Poland; agnieszka.gola@umed.wroc.pl; 2 Department of Pharmaceutical and Pharmacological Sciences, University of Padova, 35131 Padova, Italy; andreaberna@live.com (A.B.); gianfranco.pasut@unipd.it (G.P.)

**Keywords:** microparticles, 2-acrylamido-2-methyl-1-propanesulfonic acid, lower critical temperature solution, anionic initiator, potassium persulfate, electrical conductivity, controlled drug delivery

## Abstract

Thermosensitive polymers PS1–PS5 were synthesized via the surfactant free precipitation polymerization (SFPP) using 2-acrylamido-2-methyl-1-propanesulfonic acid (AMPSA), and potassium persulfate (KPS) at 70 °C in aqueous environment. The effect of KPS concentrations on particle size and lower critical temperature solution (LCST) was examined by dynamic light scattering (DLS). The conductivity in the course of the synthesis and during cooling were investigated. The structural studies were performed by attenuated total reflectance-Fourier transform infrared spectroscopy (ATR-FTIR), H nuclear magnetic resonance (^1^H NMR), thermogravimetric analysis (TGA/DTA) and powder X-ray diffraction (PXRD). ATR-FTIR, ^1^H NMR and PXRD data confirmed the polymeric nature of the material. TGA/DTA curves demonstrated thermal stability up to approx. 160 °C. The effect of temperature on the hydrodynamic diameter (HD) and zeta potential (ZP) were evaluated by dynamic light scattering (DLS) and electrophoretic mobility (EM) in 18–45 °C range. The LCST values were between 30 and 34 °C. HD and polydispersity index (PDI) of aqueous dispersions of the synthesized polymers PS1–PS5 at 18 °C were found to be 226 ± 35 nm (PDI = 0.42 ± 0.04), 299 ± 145 nm (PDI = 0.49 ± 0.29), 389 ± 39 nm (PDI = 0.28 ± 0.07), 584 ± 75 nm (PDI = 0.44 ± 0.06), and 271 ± 50.00 nm (PDI = 0.26 ± 0.14), respectively. At 18 °C the ZPs of synthesized polymers suspensions were −13.14 ± 2.85 mV, −19.52 ± 2.86 mV, −7.73 ± 2.76 mV, −7.99 ± 1.70 mV, and −9.05 ± 2.60 mV for PS1–PS5, respectively. We found that the initiator concentration influences the physicochemical properties of products including the size of polymeric particles and the LCST.

## 1. Introduction

Polymeric micro- and nanoparticles have a broad application in the pharmaceutical industry and medicine [1,2,3,4,5]. An interesting subgroup among these compounds is represented by polymers responsive to the external stimuli leading to a reversible alteration in their physical or chemical properties. There are numerous environmental factors, such as temperature, pH, light, and electric field, to which stimuli-responsive materials respond with changes in shape, volume, solubility, conductivity, and conformation [6,7]. Due to the ability to control and fine-tune the smart polymer sensitivity, they are extensively studied for controlled drug delivery systems [8]. The thermosensitive polymers have been widely and extensively studied. They are well characterized due to the relatively uncomplicated control of the stimulus via external heating or cooling [9]. Much research is also focused on pH-sensitive polymers, which may detect and react to exceptional pH changes in organs, tissues, or cells, including blood pH of 7.35–7.45 or stomach pH 1.0–3.0 [10,11,12]. In some biomedical applications, such as tumor-specific delivery, multi-responsive polymers that combine the lower critical solution temperature (LCST) with another response triggered by another factor, for example, pH, are of particular interest [13,14].

The 2-acrylamido-2-methyl-1-propanesulfonic acid (AMPSA) is an ionic monomer usually used as a comonomer to synthesize polymeric compounds exhibiting a specific response to various external stimuli. The incorporation of AMPSA units in the polymer chain was generally responsible for its pH-sensitivity, attributed to the presence of functional group –SO_3_H [15]. The AMPSA-derived polymers found different applications in many areas such as water treatment, skincare products, superabsorbents, and hydrogels [16,17,18].

The poly-2-acrylamido-2-methyl-1-propanesulfonic acid (PAMPSA), due to the presence of strongly ionized sulfonate, is widely classified as a pH-sensitive polymer; however, PAMPSA also exhibits heat-sensitive properties. The possibility of using intelligent polymers in biomedical applications requires detailed characterization of these compounds in terms of physicochemical properties and the necessity to obtain a product with strictly defined parameters, for example, particle size or electrochemical potential in controlled production [19].

This work aims to synthesize anionic homopolymers of 2-acrylamido-2-methyl-1-propanesulfonic acid and evaluate the impact of different concentrations of anionic initiator potassium persulfate on the physicochemical properties of newly synthesized polymeric products. The use of many analytical techniques for testing the properties of the obtained polymers and comparing the results may enable an accurate and reliable assessment of the final product quality and determine its suitability as an intelligent drug carrier responsive to external temperature and pH stimuli. This work focuses on the influence of starting composition and synthesis conditions on the selected physicochemical properties of aqueous suspensions and solid forms of synthesized microstructures. To the best of our knowledge, this is the first study on the synthesis of PAMPSA homopolymers using regularly varied substrate ratios and comparing the polymerization products. Previous studies on AMPSA, focused chiefly on the synthesis of combining AMPSA with other compounds supporting the polymer structure [15,16,17].

## 2. Materials and Methods

### 2.1. Materials

All chemicals and solvents were purchased from commercial and industrial suppliers and used as received without further purification or modification. The following chemicals and products were obtained from Sigma-Aldrich: 2-acrylamido-2-methyl-1-propanesulfonic acid (AMPSA, 98%, St. Louis, MO, USA), potassium persulfate (KPS, 97%, Sternheim, Germany), dimethyl sulfoxide-d_6_–NMR solvent (DMSO-d_6_, 99.96 at % D containing 0.03% tetramethyl silane-TMS, Sternheim, Germany), and dialysis tubing cellulose membrane (MWCO 3,5 kDa, Spectrum Laboratories, Inc., Rancho Dominiguez, CA, USA). Deionized water (<0.06 µS cm^−1^), applied in all following procedures, was filtered in an HLP 20 system equipped with a 0.22 µm microfiltration capsule (Hydrolab, Straszyn, Poland) and fulfilled requirements of PN-EN ISO 3696:1999 for analytical laboratories.

### 2.2. Synthesis

Five thermosensitive polymers (PS1, PS2, PS3, PS4, and PS5), in other words, 2-acrylamido-2-methyl-1-propanesulfonic acid derivatives, were synthesized via the surfactant-free precipitation polymerization (SFPP) in an aqueous system under a nitrogen atmosphere. All the experiments were performed at the monomer (AMPSA) initial concentration of ~9.15 g/L with varied anionic initiator (KPS) concentrations, ranging from 0.064 to 5.980 g/L. Table 1 presents the summary of initial conditions for synthetized PAMPSA microparticles with acronyms of the products. The polymerization was carried out in a four-necked, 2000-mL round-bottom flask (reaction vessel) fitted with an Allihn condenser (length: 300 mm), temperature sensor, conductivity cell (*K* = 1.0 cm^−1^), and magnetic stirring bar (250 rpm). The reactor vessel, immersed in a water bath, was filled with 900 mL of deionized water (solvent for all reactants, ~0.09 µS·cm^−1^ in *T* = 25 °C). The water-filled reactor was heated, under nitrogen flow, to the polymerization temperature, in other words, 70 °C. As soon as the polymerization temperature was reached, the given amount of the dry sample of the initiator was introduced to the reaction vessel. Next, after ca. 10 min of nitrogen bubbling, 100 mL of aqueous monomer (AMPSA) solution, heated to ca. 40 °C, was introduced to the reaction system to start the polymerization reaction. The total volume of the reaction mixture was 1000 mL. The polymerization reaction was carried out for 6 h at a constant temperature of 70 ± 1 °C, under nitrogen flow and continuous stirring. After polymerization, the reaction mixture was left for ca. 15 h to cool to ambient temperature. The synthesized products were purified via dialysis against stirring distilled water at room temperature to remove unreacted monomer and initiator. The purification process was as follows: ca. 170 mL of each reaction mixture (PS1–PS5) was introduced to a semipermeable dialysis cellulose tube (molecular weight cut-off: 3.5 kDa, nominal flat width: 45 mm) and placed in a 2000 mL cylinder filled with distilled water replaced once a day after measuring the conductivity. The purification process was continued for ca. 21 d and finished at conductivity close to the distilled water of ca. 1.5–2.5 µS cm^−1^. Purified aqueous dispersions of polymeric products were stored in a dark glass bottle and used to study hydrodynamic diameter and zeta potential. Samples of 100 mL of dispersion were frozen, lyophilized by Alpha 1–2 LD (Martin Christ Freeze Dryers, Osterode am Harz, Germany) two times for 26 h, and stored dry. The solid form of the polymer was used in NMR, ATR-FTIR, thermogravimetric (TG), and XRPD studies.

### 2.3. Conductivity Measurements

The CC-505 conductometer (accuracy up to 19.999 mS·cm^−1^ ± 0.1%, from 20.000 mS·cm^−1^ ± 0.25%, Elmetron, Gliwice, Poland) fitted with an EC-60 conductivity sensor with a glass housing, cell constant of *K* = 1.0 ± 0.2 cm^−1^ (Elmetron, Gliwice, Poland). The Pt-1000A temperature sensor (0–100 ± 0.35 °C) was used for measuring the conductivity of an aqueous dispersion of the reaction mixture during the synthesis and then during the cooling procedure. The conductivity and temperature sensors were placed into the reaction mixture and kept there during the synthesis and cooling procedure. The conductivity measurements during the polymerization reaction were carried out at 70 °C using manual and, while cooling, automatic compensation of temperature.

### 2.4. Attenuated Total Reflection-Fourier Transformed Infrared (ATR-FTIR) Spectroscopy Measurements

The attenuated total reflection-Fourier transform infrared (ATR-FTIR) spectroscopy was carried with a Nicolet iS50 FT-IR spectrometer (Thermo Fisher Scientific, Madison, WI, USA) fitted with a universal ATR sampling accessory and deuterated triglycinesulphate detector (DTGS). The transmission and multiple attenuated total reflection infrared spectra of lyophilized samples were recorded at ambient temperature in the spectral range from 4000 to 400 cm^−1^ utilizing 32 scans per sample collected at a resolution of 4.0 cm^−1^. The background spectra were recorded each time before measuring the sample spectrum under experimental conditions using a blank ATR and automatically subtracted from the subsequently measured sample spectrum. A small sample was deposited directly on the flat surface of the monolithic diamond crystal cell and compressed using a pressure arm. Before each measurement, the ATR element and pressure clamp were cleaned using methanol-soaked tissue paper. ATR-FTIR spectra data were analyzed using the OMNIC software (Version 9, Thermo Fisher Scientific, Madison, WI, USA).

### 2.5. Proton Nuclear Magnetic Resonance (^1^H NMR) Spectroscopy Measurements

The ^1^H NMR spectra were measured at 299.15 K on a Bruker spectrometer with the working frequency of 300 MHz (Bruker, Rheinstetten, Germany). The deuterated dimethyl sulfoxide (DMSO-d_6_, δ = 2.49) was used as a solvent. Coupling constants (J) and chemical shifts (δ) were expressed in hertz (Hz) and ppm, respectively. Samples for NMR analysis were prepared by dissolving about 10 mg of each solid and dry polymer in 7 mL DMSO-d6, without subsequent filtration or centrifugation.

### 2.6. Hydrodynamic Diameter (HD) and Polydispersity Index (PDI) Measurements

A Zetasizer Nano ZS ZEN3600 device (Malvern Instruments, Malvern, UK) was applied to perform dynamic light scattering (DLS) measurements. The selected measurement principle enabled determining hydrodynamic diameters (HD) and polydispersity index (PDI) of the aqueous polymer particle dispersion, according to the definition in ISO 13321:1996E and ISO 22412:2008 [20,21,22]. The standard red He–Ne laser with an output power of 4.0 mW, and wavelength of λ = 633 nm, was used as the incident beam. A sensitive avalanche photodiode detector (APD) placed at a 173° angle enabled the application of a non-invasive backscattering (NIBS) system. The attenuator for the laser beam adjusted the laser power during the measurement sequence by setting it automatically. An optically translucent polyacrylic disposable DTS-0012 cuvette (Malvern Instruments, Malvern, UK) was filled with 1.0 mL of the polymer dispersion and placed in a temperature-controlled cell. The sample was transparent, homogenous without any precipitation, purified by dialysis, and not diluted. The DLS measurements were recorded at every one degree with increasing temperature from 18 to 45 °C. The number of runs in one measurement was adapted automatically in the range from 10 to 100. The equilibration time before start measurements at the new temperature was 240 s. The PDI and HD were calculated based on the cumulants analysis. All presented HD and PDI data are the average of five measurements at each temperature. The Zetasizer software (version 7.10) was used to interpret data from DLS measurements; it also enabled the use and design of custom standard operating protocols ensuring repeatability of measurements.

### 2.7. Zeta Potential (ZP) Measurements

The zeta potential (ZP) was calculated following the Smoluchowski model approximation of Henry’s equation (f(Ka) = 1.5) based on measurements of the electrophoretic mobility (EM) of charged polymer particles in an aqueous dispersion. Electrophoretic mobility measurements were performed using a Zetasizer Nano ZS ZEN3600 device (Malvern Instruments, Malvern, UK) and applying the Doppler effect (laser Doppler velocimetry, LDV). The sample was measured in U-shaped plastic (polycarbonate) cuvettes, type DTS-1070, with inbuilt gold-plated copper electrodes and 0.75 mL capillary (Malvern Instruments, Malvern, UK). The results were recorded at different temperatures, ranging from 18 to 45 °C with an interval of 1 °C. The equilibration time between two adjacent measurements was 240 s. At one temperature, the measurement was repeated five times to obtain the average zeta potential. The results of EM measurements were analyzed using Zetasizer (version 7.10) software.

### 2.8. Thermogravimetric Analysis (TGA)

A TG 209 F1 Libra instrument with an automatic sample changer (ASC) (Erich NETZSCH GmbH and Co. Holding KG, Selb, Germany) was used to examine the thermal stability of the synthesized polymeric microparticles. The TG analysis was carried out at a nitrogen flow rate of 50 mL·min^−1^ under non-isothermal heating conditions. The temperature change was controlled from 25 to 800 °C at a heating rate of 5 °C·min^−1^. The samples of about 5.0 ± 0.1 mg mass were placed in opened Al_2_O_3_ crucibles and gently pressed. The lyophilized tested material had a spongy amorphous form and was not grated beforehand. The weight loss of samples was recorded automatically as a function of temperature and time with a resolution of 0.1 mg. Netzsch Proteus 7.1.0 analysis software (Selb, Germany) was used to record and analyze the TG and DTG curves.

### 2.9. Powder X-ray Diffraction (PXRD) Analysis

The powder X-ray diffraction (PXRD) measurements were performed with a Bruker D2 PHASER X-ray diffractometer (Bruker AXS, Karlsruhe, Germany) equipped with a LynxEYE detector. Ni-filtered CuKα_1.2_ radiation 1.5418 Å under constant conditions (30 kV, 10 mA) was applied. The following range was applied in the Bragg–Brentano (θ/2θ) horizontal geometry: from 5 to 70° in steps of 0.02° with scanning speed of 1.0 s/step. The variable rotation was 15 min^−1^, with a divergence slit of 1.0 mm and the shutter of 0.5 mm in size. The samples of monomers and lyophilized polymers were ground in an agate mortar and placed on a Si low-background sample holder for testing powder samples (Ø: 51.5 mm, sample cavity Ø: 20 mm × 0.5 mm). All samples were measured at 295 K and in an ambient atmosphere. Diffrac.Eva V 3.2 software was used to analyze the PXRD data.

## 3. Results

### 3.1. Synthesis

**Figure 1:**
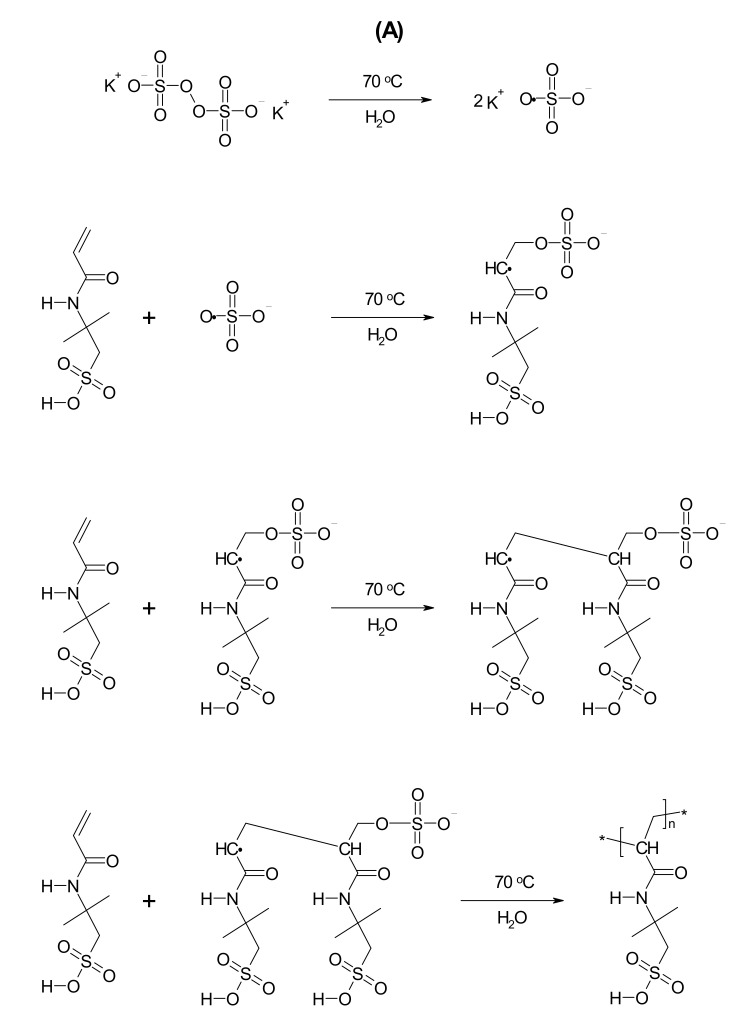

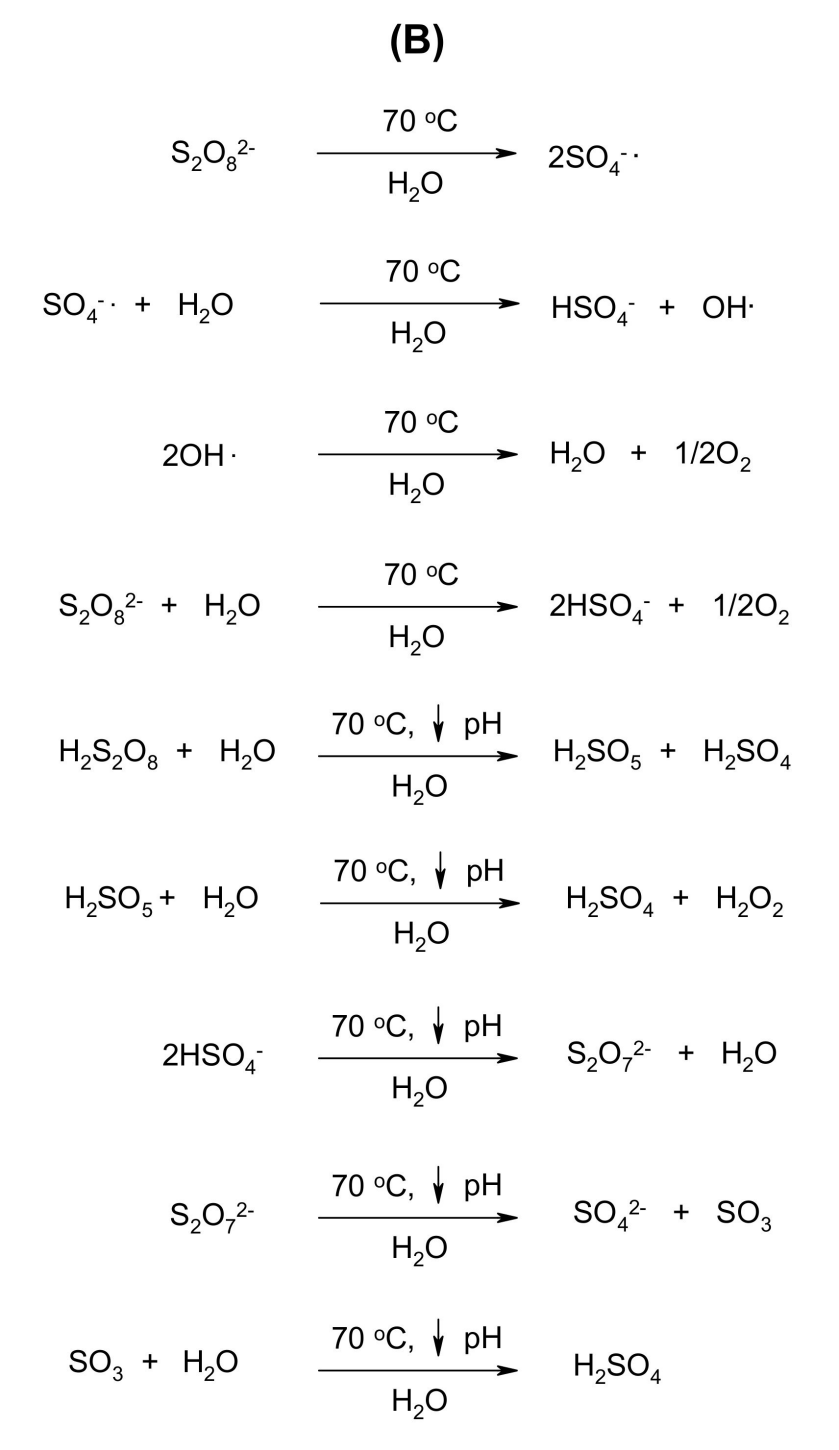
The basic steps of the synthesis of poly-2-acrylamido-2-methyl-1-propanesulfonic acid (PAMPSA) by polymerization of 2-acrylamido-2-methyl-1-propanesulfonic acid (AMPSA) aqueous solution in the presence of potassium persulfate (KPS) initiator (**A**) and potential reactions of radicals and ions produced by potassium persulfate decomposition in water (**B**).

**Figure 2:**
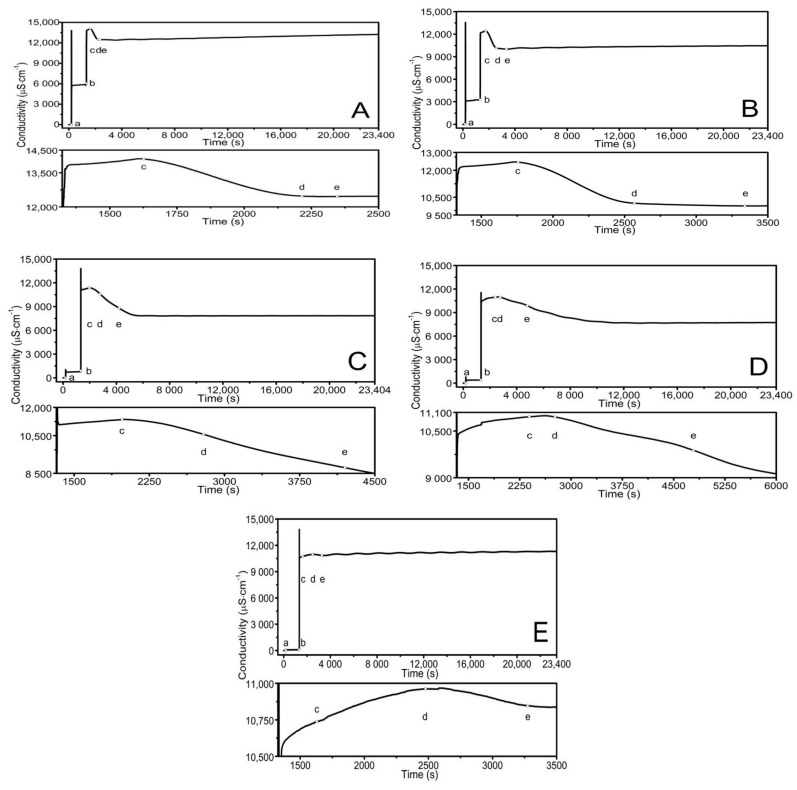
(**A**–**E**) Dependence of conductivity in the function of time in the reaction dispersion of PS1 (**A**), PS2 (**B**), PS3 (**C**), PS4 (**D**), and PS5 (**E**) in the course of the polymerization reaction at *T* = 70 °C. Point (a) marks the addition of an initiator—KPS, point (b) the addition of the aqueous solution of the monomer—AMPSA, point (c) the visible change in color of the reaction mixture, point (d) indicates the decrease in color intensity, and point (e) total disappearance of yellow color.

Polymeric PS1–PS5 microparticles, derivatives of PAMPSA, were synthesized via surfactant-free precipitation polymerization (SFPP) based on Pelton’s procedure for synthesizing poly(*N*-isopropylacrylamide) also applied in our previous studies. The polymerization processes were carried out using the same temperature, stirring rate, nitrogen atmosphere, aqueous solvent, and reaction proceeding. The syntheses differed by the initial concentration of anionic initiator, namely, potassium persulfate (KPS). The PS1–PS5 were synthesized at the following molar ratios of monomer to radical: 1:1, 1:0.5, 1:0.1, 1:0.05, 1:0.01, respectively, as presented in Table 1. The synthesis process was described in Section 2.2, Synthesis. There was no specific turbidity after the reaction initiation. However, the AMPSA in the reaction system induced a change of the initially transparent reaction liquid to yellow. The rate of color appearance and its intensity was proportional to the KPS concentration used for the reaction. After combining the initiator and monomer solutions, the noticeable color change time was found at 1626, 1757, 1909, 2390, and 1630 s in the PS1, PS2, PS3, PS4, and PS5 systems, respectively. The color disappeared with time, and reaction mixtures remained colorless until the end of the synthesis and even after the systems were cooled to room temperature. The schematic diagrams illustrating the suggested major stages of radical polymerization of 2-acrylamido-2-methyl-1-propanesulfonic acid and potential reactions of radicals and ions produced by potassium persulfate decomposition in water are presented in Figure 1 [23,24,25,26,27,28]. The color onset times were marked on the conductivity vs. time curves in Figure 2A–E as point (c).

The PS1–PS5 polymeric dispersions were purified via forced equilibrium dialysis (FED) against distilled water and then freeze-dried. As a result of these procedures, a solid, white powdery form of the synthesized polymers was obtained. The amounts of solid products from 100 mL of cleaned dispersions were 0.04530, 0.05301, 0.03950, and 0.04417 g for the PS1, PS2, PS3, and PS4 samples, respectively, while the PS5 evaporated in the freeze-drying procedure. Therefore, it was impossible to perform and present the ATR-FTIR, NMR, PXRD, and TG studies for the PS5 sample. The lyophilized samples were not stable in the ambient conditions and, over time, transformed into transparent, yellow, sticky, hardening droplets.

### 3.2. Conductivity Measurements

The electrolytic conductivity level of the reaction system was monitored during the synthesis at 70 °C and after polymerization, during cooling, in the temperature and time functions. The readings were taken every second. Figure 2A–E presents the conductivity profile of aqueous PS1–PS5 polymer dispersions over the polymerization process. The initially measured conductivity was ca. 0.722 µS cm^−1^, stable over time, and derived from the pure solvent ions. The further readings of high conductivity: 13,800, 13,560, 1058, 795, and 122 µS cm^−1^ for PS1, PS2, PS3, PS4, and PS5, respectively, resulted from the introduction of an adequate amount of powder initiator into the reaction system—Figure 2A–E, point (a). The conductivity decreased rapidly to approximately 5800, 3000, 750, 390, and 80 µS cm^−1^ for PS1–PS5, respectively, in a few seconds. Then a slightly upward trend was observed within ca. 10 min. The monomer solution addition resulted in a pronounced shift of the conductivity between 11,550 and 13,850 µS cm^−1^—Figure 2A–E, point (b). In PS1 and PS2, the rapid conductivity increase was followed by a further mild increase. In contrast, in PS3–PS5, the increase was followed by an immediate and sharp decline of conductivity, succeeded by a further gentle increase. The conductivity increased for ca. 280, 380, 660, 1270, and 1250 s for PS1–PS5, respectively. The gradual decrease of conductivity was observed until the plateau: fast for PS1 and PS2: ca. 12,460 and 10,020 µS cm^−1^ and slow for PS3–PS5 to ca. 7830, 7690, and 10,800 µS cm^−1^, respectively.

**Figure 3:**
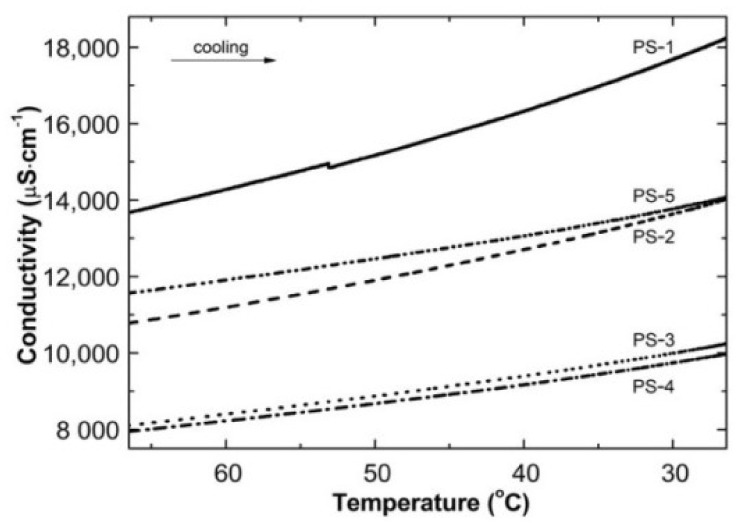
The changes of conductivity vs. temperature in the post-reaction mixtures of PS1–PS5 over cooling procedure.

**Figure 4:**
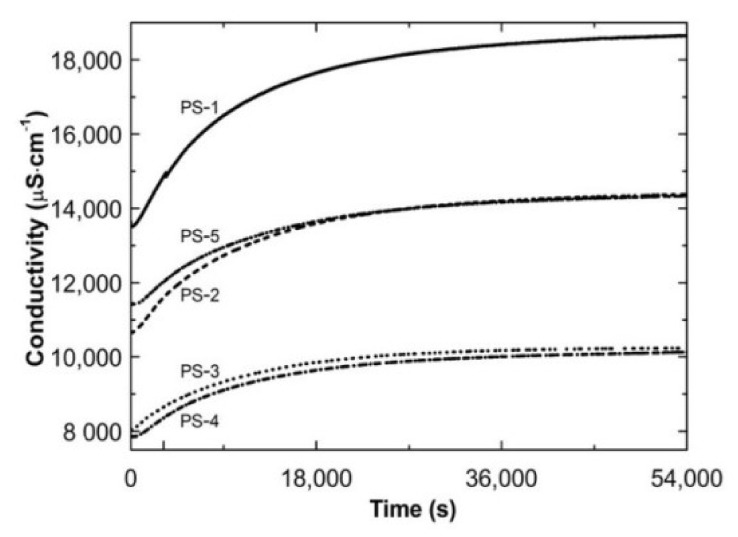
The changes of conductivity vs. time in the post-reaction mixtures of PS1–PS5 over cooling procedure.

The conductivity was also measured during cooling the polymeric systems to room temperature. Measurements were recorded versus temperature (cf. Figure 3) and time (cf. Figure 4). The tested reaction mixtures: PS1–PS5 present an almost linear dependence of conductivity as a temperature function; the conductivity systematically increased with the temperature decrease. The conductivity measurements started at ca. 68 °C, with ca. 13,641, 10,672, 8037, 7861, 11,393 µS cm^−1^ for PS1, PS2, P3, P4, and P5, respectively. The final recorded conductivities at 23.8 °C for PS1, 23.4 °C for PS2, 26.4 °C for PS3, 24.2 °C for PS4, and 23.8 °C for PS5, were 18,681, 14,392, 10,243, 10,144, and 14,337 µS cm^−1^, respectively. The estimated variability between the boundary conductivity values was 5040, 3720, 2206, 2283, and 2944 µS cm^−1^, respectively. No significant signals were found in the conductivity profiles of PS1–PS5 aqueous dispersion as a temperature function.

Figure 4 presents the conductivity of the cooled post-reaction PS1–PS5 systems in the time function. The conductivity increased exponentially until the plateau at ca. 52,679, 52,300, 50,713, 52,637, and 53,290 s for PS1, PS2, PS3, PS4, and PS5, respectively.

### 3.3. Attenuated Total Reflection-Fourier Transform Infrared (ATR-FTIR) Spectroscopy Analysis

**Figure 5:**
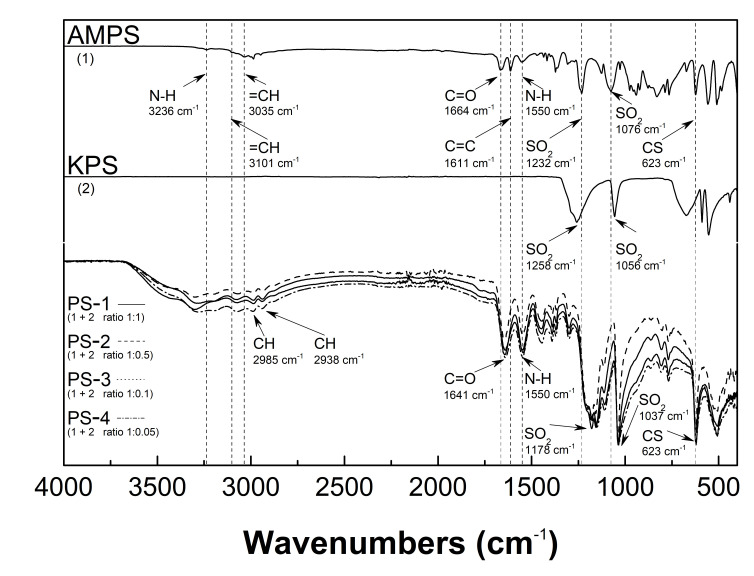
Attenuated total reflection-Fourier transform infrared (ATR-FTIR) spectroscopy: spectra of the monomer—2-acrylamido-2-methyl-1-propanesulfonic acid (AMPSA), the initiator—potassium persulfate (KPS), and synthesized PS1–PS4 homopolymers.

**Figure 6:**
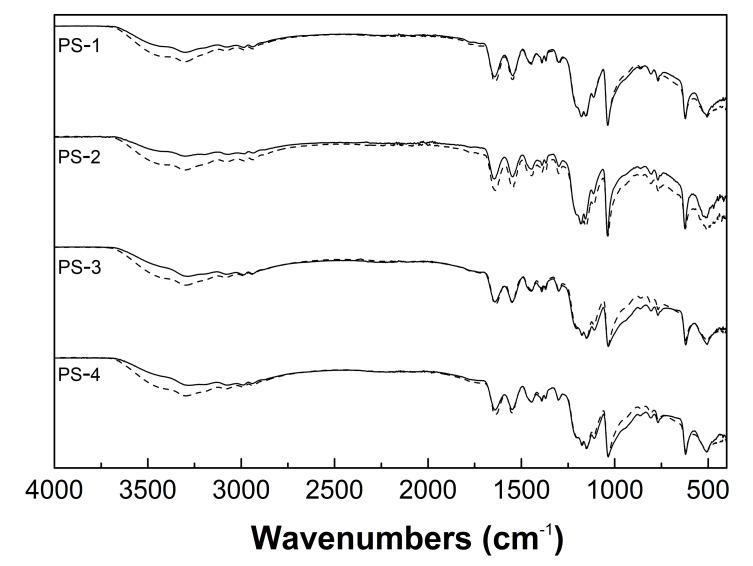
Fourier-transformed infrared spectroscopy with attenuated total reflectance (ATR–FTIR): spectra of homopolymers PS1–PS4 assessed directly after lyophilization (solid line) and after a fourteen-day exposure to the ambient atmosphere (dashed line).

The ATR-FTIR spectra of the monomer–2-acrylamido-2-methyl-1-propanesulfonic acid (AMPSA), the initiator–potassium persulfate (KPS), and synthesized PS1, PS2, PS3, and PS4 homopolymers in the transmittance mode are presented in Figure 5. Both the monomer spectrum and the sample spectra contain strong bands assigned to C=O stretching vibrations of the amide group at 1664 cm^−1^ (AMPSA) and 1641 cm^−1^ (PS1–PS5) and the N-H bending vibrations, appearing in all cases at the same wavelength of 1550 cm^−1^ [29,30,31]. Moreover, the spectrum of AMPSA presents some characteristic peaks: at 3236 cm^−1^ and corresponding to N-H [32] stretching vibrations of the amide group, at 3101, 3035, and 1611 cm^−1^ attributed to H-C=C stretching vibrations of the vinyl group [33,34,35] at 1232 and 1076 cm^−1^ characteristic for the asymmetric and symmetric stretching vibrations of the O=S=O group and additionally at 623 cm^−1^ assigned to stretching vibrations of the C-S group [36]. In PS1–PS4, the broad bands in the wavelength range 3600–3100 cm^−1^ result from the overlapping of the N-H and O-H stretching vibrations [37,38]. Two peaks appeared at positions 2985 and 2938 cm^−1^ and may be attributed to the aliphatic C-H stretching vibrations [39]. Furthermore, the stretching vibrations of O=S=O were found to be shifted to lower wavenumbers and appeared at 1178 and 1037 cm^−1^ [40]. The peak of C-S stretching vibration appeared in the polymer spectra at 623 cm^−1^ without any shifting. The ATR-FTIR spectrum of pure K_2_S_2_O_8_ salt displays two intense peaks at 1258 and 1056 cm^−1^ due to the sulfonic group [41]. Figure 6 presents the spectra of pulverized PS1–PS4 assessed directly after lyophilization and analogical exposure to water vapor of the ambient atmosphere for 14 days.

### 3.4. Proton Nuclear Magnetic Resonance (^1^H NMR) Spectroscopy Analysis

**Figure 7:**
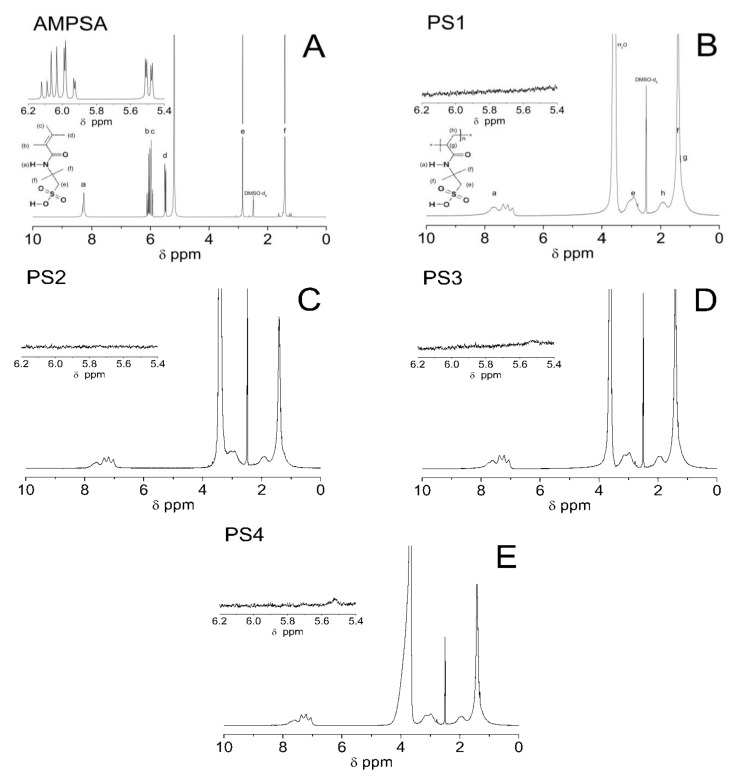
(**A**–**E**) H nuclear magnetic resonance (^1^H NMR) spectra of monomer AMPSA (**A**) and synthesized polymers PS1 (**B**); PS2 (**C**); PS3 (**D**); and PS4 (**E**) recorded in deuterated dimethyl sulfoxide (DMSO-d_6_). The expanded areas in the ^1^H NMR spectra present the resonance range of the vinyl protons.

Figure 7A–E presents ^1^H NMR spectra of monomer AMPSA and synthesized homopolymers PS1–PS4. The ^1^H NMR spectrum of AMPSA had six different peaks. The peaks were labeled in terms of structural formula as the spectra a–f were ordered from the highest value of the chemical shift. The single peak at around δ = 8.27 ppm is assigned to H_a_ proton from the NH group. The pool signals in the range of δ = 6.13–6.01, δ = 6.00–5.90, and δ = 5.54–5.44 ppm corresponded to H_b_, H_c_, and H_d_ protons from the vinyl group, respectively. In addition, the appearance of two signals ca. δ = 2.85 and δ = 1.41 ppm may be attributed to H_e_ and H_f_ protons from -CH_2_-SO_3_H and (CH_3_)_2_-C groups, respectively. There is a strong signal at δ = 5.19, which may originate from acidic hydrogen atoms—SO_3_H (Figure 7A) [42]. There are five different peaks at the characteristic chemical shifts on the ^1^H NMR spectra identified in every PS1–PS4 sample. The assigned protons were labelled in the structural formulas and the polymer spectra as a, e, f, g, h (Figure 7B–E). The signals between δ = 7.99–6.92 ppm belonged to H_a_ protons of the NH group and presumably also to protons derived from the exchange between the amide and sulfonic acid hydroxyl groups [43]. The δ = 3.3–2.6 ppm shift corresponded to the H_e_ proton from the CH_2_ group bonded to the SO_3_H group [29]. The signal of δ = 1.93 ppm revealed the H_h_ proton of the methylene group in the main chain. In other alkyl CH groups, the H_g_ protons appeared at δ ca. 1.32 ppm and were predominantly overlapped by the strong resonance signal, visible at δ ca. 1.41 ppm, assigned to H_f_ protons from equivalent methyl groups [37]. The characteristic peaks for NMR solvent, deuterated dimethyl sulfoxide (DMSO-d6), and H_2_O were observed at δ = 2.49 and δ = 3.56, respectively.

### 3.5. Hydrodynamic Diameter (HD)

**Figure 8:**
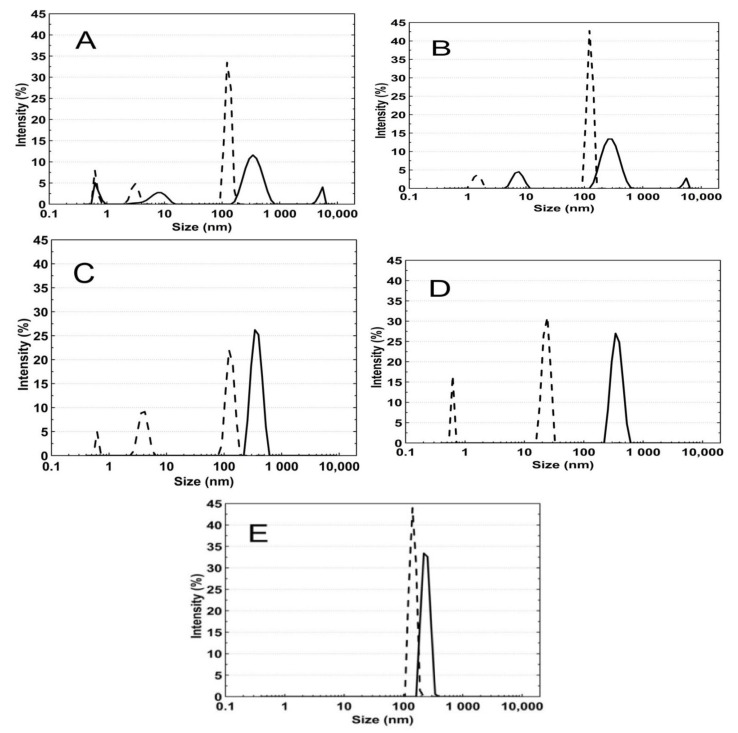
**(A**–**E**) Intensity size distribution curves for the PS1 (**A**), PS2 (**B**), PS3 (**C**), PS4 (**D**), and PS5 (**E**) dispersions at 18 °C—solid line and 45 °C—dash line, determined by dynamic light scattering.

In order to determine the hydrodynamic diameter (HD) and thermo- and pH-responsivity of the synthesized polymers, the dynamic light scattering (DLS) technique was applied. The HDs in the tested group of PS1, PS2, PS3, PS4, and PS5 microstructures assessed at 18 °C were 226 ± 35 nm, 299 ± 145 nm, 389 ± 39 nm, 584 ± 75 nm, and 271 ± 50 nm. At 45 °C, the HD achieved bigger sizes: 807 ± 549 nm, 812 ± 344 nm, 639 ± 333 nm, 2915 ± 1876 nm, and 592 ± 92 nm, respectively. Figure 8A–E shows distribution curves for PS1–PS5 samples at 18 °C and 45 °C. The size data were obtained from the calculations according to the Stokes–Einstein equation.

#### 3.5.1. Thermo-Responsivity

**Figure 9:**
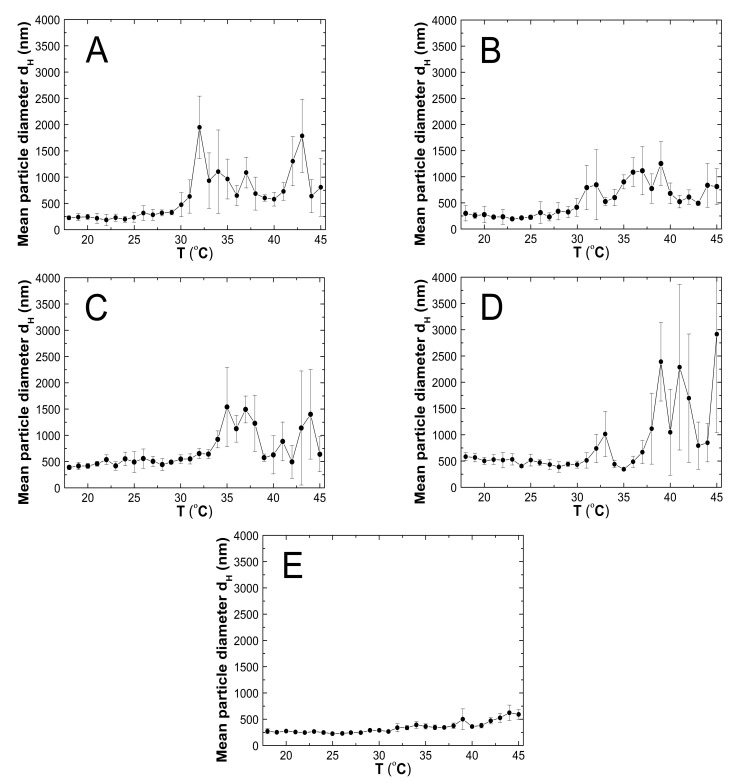
(**A**–**E**) The influence of temperature on the hydrodynamic diameter of the PS1 (**A**), PS2 (**B**), PS3 (**C**), PS4 (**D**), and PS5 (**E**) samples, determined by dynamic light scattering.

The HD measurements of PS1–PS5 purified aqueous suspensions were carried out at a temperature ranging from 18 to 45 °C (Figure 9A–E). Regardless of the initiator concentration, the HD vs. temperature plots exhibited similar profiles, however, on a different scale. The HDs remained stable with slight deviations up to 30 °C for PS1 and PS2, 33 °C for PS3, 35 °C for PS4, and 31 °C for PS5. A temperature increase of 1–2 °C over the above ranges resulted in a sharp increase of HD, corresponding to phase transition. The HD of P1, P2, P3, P4, and PS5 polymers quickly increased from 475 to 1945 nm: in the 30–32 °C range, from 415 to 846 nm: in 30–32 °C, from 643 to 1540 nm: in 33–35 °C, from 344 to 2391: in 35–39 °C, and from 266 to 392 nm: in 31–34 °C, respectively. Over the phase transition temperature, HD fluctuations were quite large and affected by a high standard error.

#### 3.5.2. The pH-Responsivity

Table 2 lists the values of hydrodynamic diameters of the PS1–PS5 samples studied in five different pH buffers, ranging from pH 3 to 7 at 25 °C. Furthermore, 0.5 mL of the purified polymer dispersions were placed into polystyrene disposable cuvettes filled with 0.5 mL of the buffer solution with different pH. The effects of pH on the size of PS1–PS5 polymer microparticles were investigated 24 h after sample preparation at 25 °C. All measurements were repeated three times.

The hydrodynamic diameter data obtained in buffers at different pH values indicate that changes in size are more visible in the buffers at pH range 5–7 than in the range 3–5. The individual samples of PS2–PS5 polymers represented similar HD in buffers at the pH range 3–5, while a rapid increase occurred at the pH ca. 6.

The exception is the PS1 sample: the hydrodynamic diameters systematically increased in buffers at pH range 3–5, in buffer at pH 6, a decrease was observed; the environmental pH of 7 resulted in an increase of the hydrodynamic diameter.

### 3.6. Polydispersity Index (PDI)

**Figure 10:**
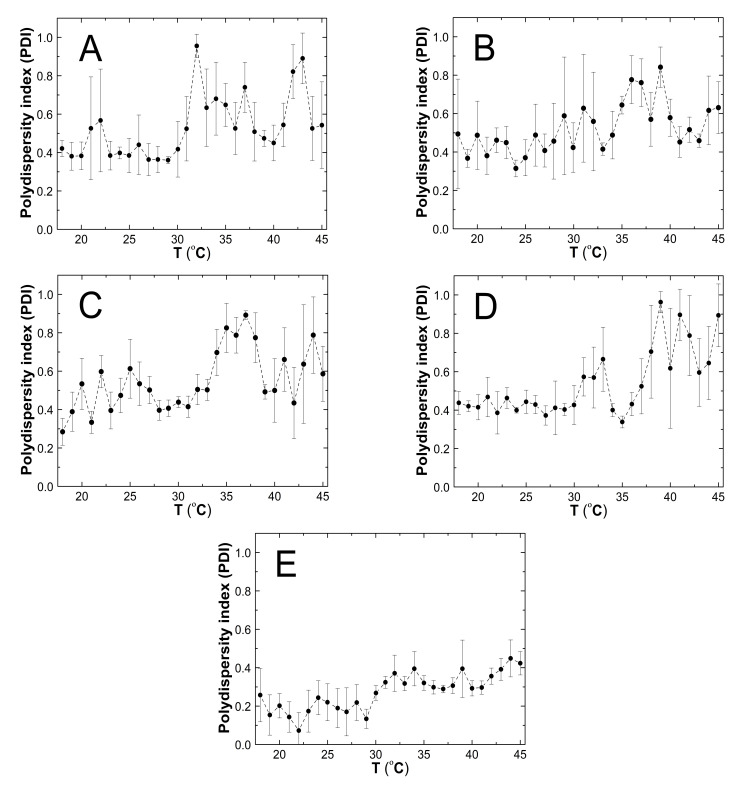
(**A**–**E**) The influence of temperature on the polydispersity index (PDI) of the PS1 (**A**), PS2 (**B**), PS3 (**C**), PS4 (**D**), and PS5 (**E**) samples, determined by dynamic light scattering.

The influence of temperature on polydispersity index (PDI) of synthesized polymeric particles was investigated in the temperature range from 18 to 45 °C in aqueous dispersions—Figure 10A–E. The polydispersity remained approximately stable between 18 and 30 °C for assessed samples; the average PDI value range was 0.41 ± 0.11, 0.44 ± 0.14, 0.45 ± 0.08, 0.42 ± 0.07, and 0.19 ± 0.09 for PS1, PS2, PS3, PS4, and PS5, respectively. A remarkable PDI increase was observed with temperature increase. The PS2–PS5 samples revealed a maximum with a consequent slight decrease of PDI, which remained at a predominantly constant level with slight deviations. The PS1 presented two peaks of PDI (Figure 10A) of 0.96 ± 0.06 and 0.89 ± 0.13, at 32 °C and 43 °C, respectively. Over these temperatures, a slight PDI decrease was observed to 0.60 ± 0.13 (33–42 °C) and 0.53 ± 0.20 (44–45 °C), respectively.

PS2–PS5 achieved one PDI maximum of 0.84 ± 0.10 at 39 °C, 0.89 ± 0.02 at 37 °C, 0.96 ± 0.06 at 39 °C, and 0.39 ± 0.09 at 34 °C, respectively—Figure 10B–E. The temperature increase up to 45 °C did not influence particular changes in PDI. The estimated average PDI values for PS2 in the 40–45 °C range were 0.54 ± 0.10, for PS3 in 38–45 °C—0.61 ± 0.17, for PS4 in 40–45 °C—0.74 ± 0.20, and for PS5 in 35–45 °C—0.35 ± 0.06.

### 3.7. Zeta Potential (ZP)

**Figure 11:**
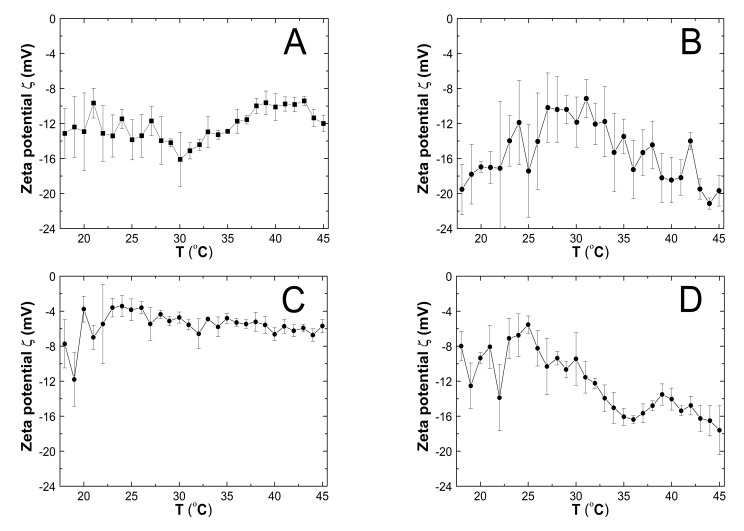

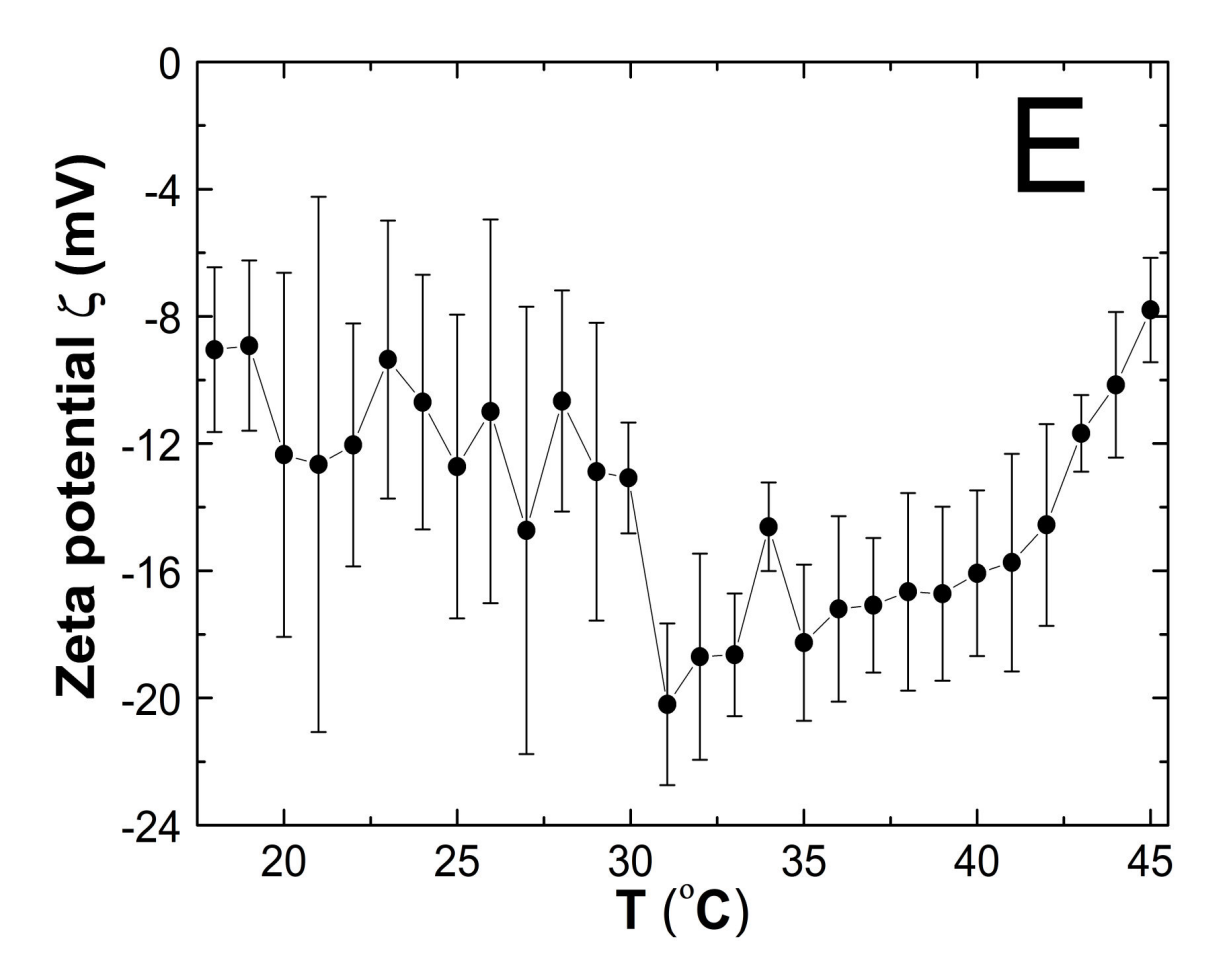
(**A**–**E**) The influence of temperature on zeta potential (ZP) of the PS1 (**A**), PS2 (**B**), PS3 (**C**), PS4 (**D**), and PS5 (**E**) samples, determined by electrophoretic mobility.

The zeta potentials (ZPs) of the PS1–PS5 aqueous dispersions were recorded in the range from 18 to 45 °C (Figure 11A–E). The pH values of PS1, PS2, PS3, PS4, and PS5 purified unbuffered samples were acidic: 3.3, 3.7, 3.24, 3.34, and 5.3, respectively, at ca. 22.5 °C. The ZPs measured in the entire assessed temperature range were negative. The smallest module ZP values, ranging from −11.8 to −3.42 mV in a temperature from 18 to 45 °C, were recorded for the PS3 sample. In the remaining cases, the zeta potential varied from −16.10 to −9.42 mV for PS1, from −21.14 to −9.14 mV for PS2, from −17.60 to −5.54 mV for PS4, and from −20.20 to −7.80 mV for PS5. The highest absolute value of ZP at 18 °C was observed for sample PS2—19.52 mV. Moreover, the PS1, PS2, and PS5 ZPs measured at 18 °C and 45 °C were the closest to each other: −13.14 and −12.00 mV, −19.52 and −19.58 mV, and −9.05 and −7.80 mV, respectively. In comparison, the differences between the zeta potential values for PS3 and PS4, achieved at the initial and final measurement temperatures, were greater and amounted to approximately 35 and 55%. Following the ZP temperature trends (Figure 11A–E), the temperature of 30 °C and higher seems critical as an onset indicator of the pronounced changes in the ZP of PS1–PS5. With the temperature increase to 30 °C, the ZP values changed with a large fluctuation, without definitive evidence of an upward or downward trend, except for PS2. For the PS2 polymer, an increase in temperature from 18 to 30 °C resulted in a gradual increase of zeta potential from −19.52 to −11.85 mV. Above 30 °C, as the temperature increased, the ZP values decreased subtly for the PS3 polymer and significantly for PS2 and PS4. However, for the PS4 polymer, during the downtrend, two distinct changes were noted. Initially, the ZP value gradually decreased to −16.38 at 36 °C, next increased to −13.52 at 39 °C, and then decreased again to −19.48 at 45 °C. In the case of PS1, over 30 °C, there was a regular increase to −9.42 at 43 °C, followed by a rapid decline of ZP to −12.00 at 45 °C. In the temperature range from 30 to 45 °C, PS5 exhibited steadily rising ZP.

### 3.8. Thermogravimetric Analysis (TGA)

**Figure 12:**
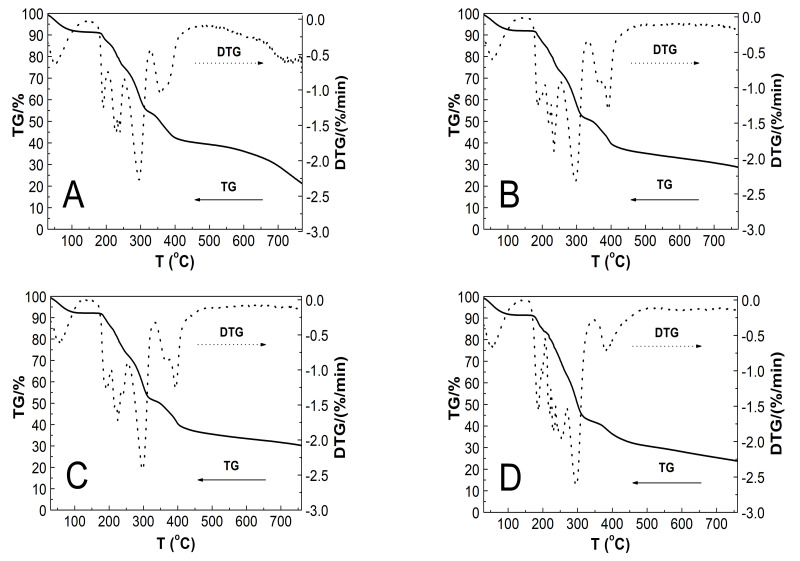
(**A**–**D**) TG—solid line, and DTG—dashed line, plots of weight loss (%) vs. temperature of polymers, PS1 (**A**), PS5 (**B**), PS3 (**C**), and PS4 (**D**). The heating rate was 5 °C min^−1^ in a nitrogen atmosphere at 50 mL min^−1^.

The characteristic TG and DTG plots of the thermal decomposition of microspheric homopolymers, PS1–PS4, with the decreasing monomer to initiator weight ratio, obtained at a heating rate of 5 °C·min^−1^ in an N_2_ atmosphere are presented in Figure 12A–D. The thermal stability of tested polymers was characterized from 30 to 760 °C and presented as a percentage of mass loss.

The samples presented a similar thermal degradation profile with three stages of weight loss. The first stage was observed at 30–110 °C range, with weight loss of ca. 7.53, 7.79, 7.13, and 7.74% for PS1, PS2, PS3, and PS4 polymers, respectively. The second stage entailed a major weight loss of ca. 37.24% for PS1, 40.79% for PS2, 40.78% for PS3, and 48.35% for PS4, which occurred between 160 and 330 °C. The third stage of weight loss was 13.70, 15.16, 15.04, and 11.29% for PS1, PS2, PS3, and PS4, respectively, between 335 and 480 °C. The characteristic thermal decomposition data of all four tested homopolymer types, determined by TGA, are presented in Table 3. *T*_Onset_ is the extrapolated onset temperature at which degradation starts; *T*_1_, *T*_2_, and *T*_3_ are the peak temperatures in the DTG graphs corresponding to the decomposition temperature visible as weight loss stages at TG; *T*_Endset_ is the extrapolated temperature at which the degradation process is finished; *T*_1.0wt%_ is the temperature at which 1.0 wt% loss occurred, and Res the percentage of residual mass estimated at 760 °C.

### 3.9. Powder X-ray Diffraction (PXRD) Analysis

**Figure 13:**
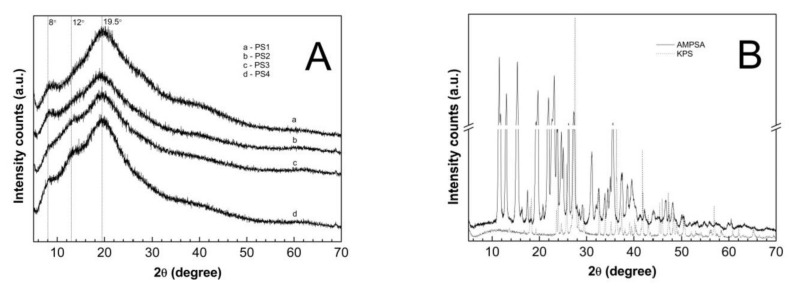
(**A**,**B**) Powder X-ray diffraction patterns of (**A**) synthesized PS1–PS4 polymers; (**B**) monomer–AMPSA and initiator–KPS.

The wide-angle PXRD diffraction patterns of the lyophilized and pulverized PS1–PS4 samples, as well as monomer and initiator samples, are demonstrated in Figure 13A,B, while the respective intensities are presented in Table 4.

The diffraction patterns contain an amorphous halo with two explicit peaks at approximate reflection angles. The dominant, broadest, and strongest maximum at ~19.50° 2θ and the smaller one at ~8.00° 2θ were detected. There were discernable differences in the intensity of the diffraction peaks at the reflection angle ~8.00° 2θ. The height and width of the peaks for PS1 and PS2 were similar and significantly higher than for PS3 and PS4. In addition, the diffraction lines for PS3 and PS4 have no pronounced peak at ~12.00° 2θ, absent in the diffraction patterns of PS1 and PS2. Figure 13B presents the PXRD profiles of the commercial samples of monomer AMPSA (solid line) and initiator KPS (dotted line). The PXRD patterns of AMPSA and KPS exhibit sharp and intense crystalline peaks in the range of 5–70° 2θ and 17–70° 2θ, respectively. The strongest crystalline peaks for the AMPSA sample are at 2θ values of ca. 11.50°, 15.30°, and 23.10°. The diffractogram of KPS presents one major peak, definitely prevalent over the others at ca. 27.50° 2θ. Moreover, between 6.01 and 16.05° 2θ no extra sharp peaks were detected. In mentioned reflection angle range, a broad, flat peak appears with a maximum at 2θ of 11.30°.

## 4. Discussion

### 4.1. Synthesis

In this study, five thermo- and pH-sensitive homopolymers were synthesized using decreasing KPS initiator concentrations, namely, PS1, PS2, PS3, PS4, and PS5. The resulting polymer network is formed of the 2-acrylamido-2-methyl-1-propanesulfonic acid. The direct product of the synthesis is an aqueous dispersion of polymers. The linearity and high solubility of polymer structures resulted in transparent mixtures. The temperature of 70 °C guaranteed effective decomposition of the initiator into two twin free radicals. Although in our case, more different radicals (Figure 1) could have been formed in the reaction system due to the favorable reaction conditions, including the temperature and acidity [23]. The polymerization mechanism involves a number of basic elementary processes, and various kinetic relationships characterize each polymerization stage. The additional reactive species in the system may induce side reactions, disrupt the polymerization initiation process, and reduce fractions of favorable free radicals that attach to the monomer. These phenomena may limit polymer chain growth reactions. In addition, in a strongly acidic environment, a high probability exists of decomposition of potassium persulfate according to the non-radical mechanism with the formation of SO_4_ giving Caro’s acid [44]. The introduction of the monomer solution into the system did not result in characteristic macroscopic changes in turbidity during the process. However, the reaction mixtures will turn from colorless to a clear, slightly yellow, disappearing over time. Due to the presence of many components in the aqueous phase, for example, polar species and both reducing and oxidizing agents, possibly interacting, the polymerization process may be complex. Transient sulfur compounds may form to give observed temporary yellow coloration of the solution. The lyophilization of purified aqueous polymer dispersions of PS1–PS4 resulted in meager amounts of a solid amorphous product in the range of 45 mg per 100 mL of the dispersion, while the freeze-drying of PS5 was ineffective.

### 4.2. Conductivity

The consecutive stages of radical polymerization may differ in terms of reaction rate, thermal effects, or various physicochemical properties. The tracking of electrical conductivity in the course of free radical polymerization enables the approximation of the onset and duration of the individual steps of the reaction [35,45,46]. Frequent recording of electrical conductivity values enables close control of alterations in the reaction system.

The first rapid increase in conductivity was related to introducing a definite dry portion of the initiator into the reaction system and high local concentration of the ingredient in the solvent (Figure 2A–E). The initiator dose influences the recorded values of conductivity.

The following conductivity values remain constant, with a slight uptrend during the initiator decomposition into primary free radicals. The process occurs due to the kinetics of the first-order reaction, and the decay rate constant is in the order of 10^−4^–10^−6^ s^−1^ [47]. The forming radicals, surrounded by solvent molecules, may undergo the cage effect: the diffusion of radicals is impeded and facilitates the recombination reaction. Moreover, the reaction of the radical with the solvent may decrease the level of the starting initiator due to the formation of other products, for example, HSO_4_^−^, resulting in a slight increase of conductivity.

The occurrence of the gaseous phase is observed in the polymerization course. It may be ascribed to the presence of oxygen particles. The reaction is based on the hydroxyl radicals as products of peroxysulfate radicals reacting with water. The resulting hydroxyl radicals lead to the formation of oxygen molecules.

Another sharp increase in conductivity was recorded after introducing the aqueous monomer solution into the system. The polymerization reaction was initiated by attaching free radicals to the monomer molecule to form oligoradicals. The cage effect can slow down the oligoradical formation process. Moreover, the resulting oligoradicals can be surrounded by unreacted monomer molecules and solvents and stabilized by electrostatic interactions. This phenomenon may be reflected by conductivity values recorded at a relatively constant level, represented by the horizontal line approximately after the “b” and ahead of “c” in Figure 2A–E.

The next recorded sharp drop in conductivity may be attributed to the chain growth process, which consists of the gradual attachment of successive monomer molecules to the growing macroradical, approx. between “c” and “d.” The following stage achieved by the systems is a plateau state, where we did not register changes in the conductivity values. This may indicate no changes in the substrate or product concentrations and the polymerization process completion.

The profile of changes in conductivity over time at a constant temperature of 70 °C in the first stages of polymerization was very similar in the tested PS1–PS4 systems. The exception is the PS5 system, in which, compared to other systems, there are no clearly exposed changes in the conductivity value reflecting the individual stages of polymerization. Several factors may lead to insufficient initiator reactivity, including solvent excess and side reactions, which add to the already very low concentration of the starting initiator. Some small-sized polymer products are formed, which confirmed the DLS measurements and the lack of product after freeze-drying.

Contrary to our previous research on N-vinylcaprolactam [46], the analysis of diagrams in Figure 3 showed that the conductivity of the post-reaction mixture increased with the decrease in temperature. This phenomenon may result from the presence of unreacted initiator particles in the system and specific decomposition routes of the persulfate anion, which can be further studied [44,48].

The cumulative amounts of new ions formed in the post-reaction dispersion during cooling recorded as changes in the mixture conductivity over time constitute an increasing exponential function, as presented in Figure 4.

The recorded data of changes in conductivity over time can be used for the initial estimation of the kinetics of the polymerization process.

### 4.3. ATR-FTIR

The characteristic ATR-FTIR absorption bands of PS1–PS4 polymers, synthesized under different initiator concentrations, appear in the same spectral regions. This confirms the similarity of the qualitative composition of polymerization products. The comparative studies of monomer and PS1–PS4 spectra confirm as well the completion of the polymerization reaction. First of all, the monomeric bands observed at 3101, 3035, and 1611 cm^−1^, representing the stretching vibrations of unsaturated C=C bond, disappeared in the product spectra. The C-S stretching vibrations in both monomer and polymer spectra at the same wavelength of 623 cm^−1^ also confirm the polymerization occurrence. Interestingly, the ATR-FTIR spectra of synthesized polymers recorded immediately and a few days after freeze-drying differed only in terms of increased intensity of the broadband located in the range of 3600–3100 cm^−1^. The broadening of these signals may be related to the water adsorbed from the environment and the formation of intramolecular hydrogen bonds. This may indicate that the change in the form of the solid products obtained as a result of freeze-drying from powdery white to glassy yellow is the result of the high hygroscopicity.

### 4.4. ^1^H NMR

The basic product characterization in terms of vinyl bond disappearance in polymerized materials PS1–PS4 compared to the AMPSA substrate via ^1^H NMR is shown in Figure 7A–E. The multiplet attributed to the H_b_, H_c_, and H_d_ protons of the vinyl group in monomer appeared in the range δ = 6.13–5.44 ppm in the monomer spectrum not present in the product spectra. Furthermore, the expanded areas in the ^1^H NMR spectra of PS1–PS4 polymers confirmed the absence of residual peaks in the resonance range of vinyl protons. This indicates the absence of monomer units in the freeze-dried products. Moreover, PS1–PS4 spectra contained wide and intense peaks at ca. δ = 1.93 and δ = 1.32, characteristic for signals of protons of CH and CH_2_ from the polymeric chain. The ^1^H NMR spectra confirm the formation of macromolecules in the free radical polymerization reaction.

### 4.5. HD

**Figure 14:**
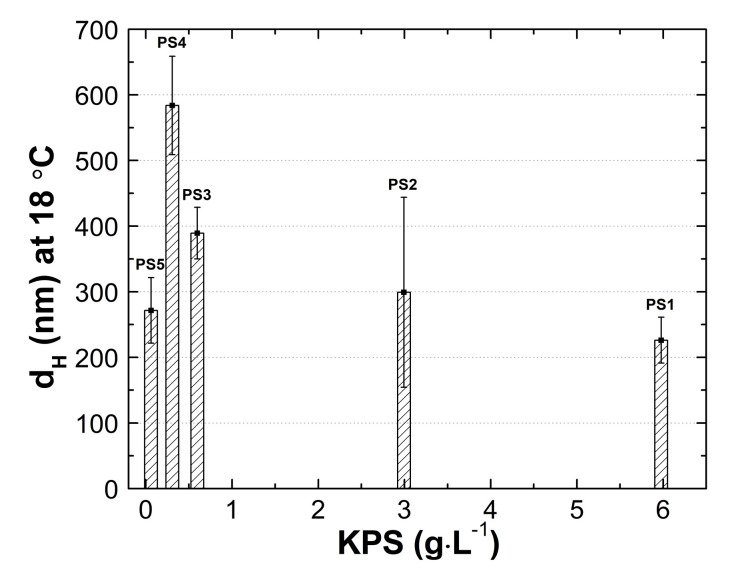
Hydrodynamic diameters of PS1–PS5 as a function of the concentration of anionic initiator, potassium persulfate (KPS).

As demonstrated in Figure 14, there is an evident influence of initiator concentration on the size of the hydrodynamic diameter of polymer particles—increased initiator concentrations favor the low hydrodynamic diameter. An exception to this rule is the PS5 polymer synthesized with the least amount of initiator. In line with the trend in PS1–PS4 polymers, the PS5 polymer should have the largest size. Although, the size of the hydrodynamic diameter of PS5 is comparable to the diameter of the PS1 and PS2. Numerous factors, including reaction medium viscosity, reaction temperature, propagating macromolecule structure, or interaction of all reaction system components, influence the macromolecule size. Here, reactions with radicals resulting from the initiator decomposition and the high acidity of the reaction medium, assured by the monomer and the initiator, may be an additional determinant of the particle size.

#### 4.5.1. Thermo-Responsivity

According to the graphs in Figure 9A–E, the obtained PS1–PS5 are temperature-sensitive polymers with lower critical temperature solution (LCST). The PS1–PS4 polymers change their hydrodynamic diameters at 30 °C, and the PS5 at 31 °C. The synthesized PS1–PS5 above the LCST showed non-typical behavior. Their hydrodynamic diameters increased in opposition to the known descriptions of the negative temperature-sensitive polymers [44,49]. We have encountered a similar phenomenon in our previous researches [35,45,46]. The increase in hydrodynamic diameter above the LCST can result from intramolecular aggregation. This may indicate that the polymeric particles rearrange during volume phase transition and an aggregation process occurs due to soft interaction forces. It can indicate that in the studied system, interactions between polymer-polymer, polymer-solvent, and between monomer-monomer included in the polymer chain through the Lennard-Jones potential, may have occurred [50,51,52]. The results show no relationship between the initial amount of initiator used for the synthesis and the temperature of the phase transition acquired by the product.

#### 4.5.2. pH-Responsivity

Table 2 presents the hydrodynamic diameters of PS1–PS5 polymers as pH function. The hydrodynamic diameters in an aqueous environment are slightly affected by pH in the range of 3–5 and significantly at higher pH values. The polymer dispersions purified via forced equilibrium dialysis had a low pH value of ca. 3.3, except 5.3 for the PS5. The pKa of monomer AMPSA is 5.71 higher than pH values of purified polymer dispersions PS1–PS5. The increase in the hydrodynamic diameter of the tested polymer systems placed in buffer mediums of pH 5–7 is a response to an increase of external pH above the pKa. In pH below 5.71, the anionic groups are protonated, and the repulsive electrostatic interactions between the protonated –SO_3_H groups are relatively slight. Contrary, in pH above the pKa, the SO_3_H groups dissociate, and the electrostatic repulsion forces between ionized negative internals groups lead to an increase in the overall dimensions of the polymer [52,53,54,55]. This effect may be enhanced by the presence of terminal groups derived from the potassium peroxide initiator in the macromolecule.

### 4.6. PDI

The PDI variability of synthesized polymers at increasing temperature may be divided into two sections: below and above the 30 °C (Figure 10A–E). The PDI values were also lower at lower temperatures and did not exceed 0.65, except PS5, which gave a value below 0.2. It indicates 1:0.05 monomer to initiator ratio as favorable in terms of size homogeneity. Interestingly, PS4 was affected by the lowest standard error in this temperature range. The considerable growth of PDI by 96–130% is related to the phase transition that occurs in the tested samples in a narrow temperature range over 30 °C. Above the estimated LCTS in all cases, the polydispersity index increased with temperature increase. It oscillated, affected by a large standard error, which indicates the formation of unstable particle systems of various sizes in PS1, PS2, PS3, PS4, and PS5 samples. The relatively high and variable PDI values, affected by a high standard error, may result from applying the free radical polymerization process, which insufficiently controls the chain termination reactions. Despite this, considering the obtained PDI values, ranging from 0.26 to 0.49 at 18 °C, the obtained polymer microparticles may be classified and accepted as compounds for the development of drug delivery systems [56].

### 4.7. ZP

The zeta potential in the pharmaceutical and biotechnological industries is often applied to assess dispersion stability. The electrostatic charge repulsion or attraction between particles is essential for aggregation or flocculation phenomena usually observed below modulus value of 30 mV [57,58]. The negative charge of PS1–PS5, measured between 18 to 45 °C, ranged from −21.5 to −3.5 mV. It was generated by ionized sulfonate groups of AMPSA units and terminally located sulfate groups from the KPS initiator. The negative surface charge may favorably increase the affinity of our synthesized potential polymeric drug carrier to the cationic drugs and the confirmed by the ATR-FTIR hydrophilicity of the macroparticles. Based on the obtained ZP results, the stability of the PS3 system can be described as rapidly coagulating and flocculating, while in the case of PS1, PS2, PS4, and PS5 as incipient instability. Consequently, the investigated dispersion systems are composed of molecules with a relatively high aggregation potential. However, at 18 °C, the PS1 and PS2 polymers, with the highest monomer to initiator molar ratios of 1:1.0 and 1:0.5, respectively, displayed the greatest negative charge. This observation could suggest that the HD of PS1 and PS2 polymers will be greater than that of PS3–PS5 polymers, but HD measurements do not confirm this pattern. Thus, the surface charge density and charge arrangement may play an important role [59,60]. The zeta potential of the investigated polymer dispersion systems depends on the temperature (Figure 11A–E). Over the phase transition temperature, systemic zeta potential changes are observed; the decreasing trend is for PS2, PS3, and PS4 systems and the increase for PS1 and PS5. The monomer to initiator molar ratios of 1:0.5 and 1:0.05 favor the stability increase with increased temperature. The PS2 molar ratio at 44 °C is 1:0.5, which results in the highest absolute value of the zeta potential and the greatest dispersion stability.

### 4.8. TG

The TG and DTG curves of microspheric homopolymers (Figure 12A–D, Table 3) indicated similar pyrolysis behavior of tested PS1–PS4 polymers. They exhibited thermal stability up to approx. 160 °C. The thermograms demonstrated three stages in the mass-loss profile. The first one covers the temperature range from 30 to 110 °C. The several percent weight loss may be attributed to moisture desorption, crystallization water release, or first-order phase transitions, for example, melting. At 160–330 °C, the following stage is ascribed to degradation, confirmed by a high percentage of weight loss. The remarkable weight loss may be attributed to the sulfonic acid group splitting-off and degradation to SO_2_ and SO_3_. At 335–480 °C, the final stage marks the actual chain degradation and decomposition of the polymer backbones.

The DTG curves also underline the sample changes, leading to a clear separation of the degradation steps. The slight protrusions on the DTG plots reflecting the weight loss may indicate other overlapped thermal processes, for example, adsorption of gaseous decomposition products on the surface of the newly formed phase. Moreover, DTG data confirmed the influence of high initial initiator concentration on the thermal stability of PS1, PS2, PS3, and PS4 polymers and the shifting of the decomposition temperature of the major mass loss stage toward higher values.

### 4.9. PXRD

The X-ray diffraction patterns of four synthesized polymers, commercial monomer, and initiator are given in Figure 13A,B. The analysis of the monomer and initiator diffractograms, presented in Figure 13B, confirmed a high degree of crystallinity of these substances, as evidenced by clear and sharp diffraction peaks. The PXRD of PS1, PS2, PS3, and PS4 did not display any sharp crystalline peaks; only two wide, blurred diffraction haloes were present. This indicates the amorphousness of the synthesis products. The X-ray patterns of tested polymers did not reveal any shifts ascribed to various amounts of initiator in the process. The decrease of initiator concentration resulted in the decreased intensity of the smaller maximum at ~8.00° 2θ, for the PS1, PS2, and PS3, respectively. However, for PS4, there was an increase in peak intensity. The peak at ~12.00° 2θ observed in PS3 and PS4 samples may indicate traces of unreacted monomer in lyophilized material; the monomer has a peak at 11.52° 2θ, and is incompletely removed in dialysis purification.

## 5. Conclusions

In this study, as a result of the surfactant free-precipitation polymerization in an aqueous environment at 70 °C, five dual temperature- and pH-sensitive homopolymers of 2-acrylamido-2-methyl-1-propanesulfonic acid, namely, PS1, PS2, PS3 PS4, and PS5, were obtained. The performed syntheses differed only in the initial initiator concentration. The influence of KPS concentration on the physicochemical properties of the obtained polymers was investigated. It was found that conductometric measurements during synthesis can be useful in monitoring the polymerization process and assessing the polymerization steps. Based on the hydrodynamic diameter studies, it was shown that the HD of the obtained polymer particles at 18 °C were in the range from 226 ± 35 to 584 ± 75 nm; and in the case of PS1–PS4, the higher the initial initiator concentration, the smaller the size of the polymer particle. Furthermore, the studies of HD changes versus temperature led to estimate the LCST between 30 and 34 °C. No relationship between the initiator concentration and the LCST value was observed. The hydrodynamic diameter measurements as a function of pH revealed a significant increase in HD in the pH range 5–7. The recorded PDI values at 18 °C, ranging from 0.26 to 0.49, indicated a relatively low polydispersity degree. The results of ZP measurements of the purified colloidal polymer particles showed the negative charge on the surface of PAMPSA particles in the temperature ranging from 18 to 45 °C and an increase in colloidal stability at higher temperatures.

The ATR-FTIR and ^1^H NMR results indicated that the polymerization reaction was due to the process of breaking the carbon-carbon double bond. Analysis of the ATR-FTIR spectra of the polymers exposed to moisture from the ambient atmosphere showed that only the O-H stretch peak changed, namely, increased. This finding confirms the marked absorption of water by the polymers. The solid polymer products were also characterized using TG and PXRD measurements. The TG tests enabled establishing the thermodynamic parameters of PS1, PS2, PS3, and PS4 polymers and stating that increased initial content of initiator in synthesis reaction leads to shifting of the decomposition temperature of the major loss stage toward higher values. The PXRD measurements of PS1, PS2, PS3, and PS4 polymers indicate that synthesis products are in an amorphous state.

## Figures and Tables

**Table 1:** Compositions of PS1, PS2, PS3, PS4, and PS5 microparticles and the molar ratio of monomer to sulfate radicals.

Type of Polymer Microparticle System	Monomer (g)	Anionic Initiator (g)	AMPSA to KPS Radical Molar Ratio
	AMPSA ^1^	KPS ^2^	
PS1	9.1520	5.9771	1:1
PS2	9.1651	2.9937	1:0.5
PS3	9.1570	5.974 × 10^−1^	1:0.1
PS4	9.1592	3.076 × 10^−1^	1:0.05
PS5	9.1563	6.081 × 10^−2^	1:0.01

^1^ AMPSA-2-acrylamido-2-methyl-1-propanesulfonic acid; ^2^ KPS—potassium persulfate.

**Table 2:** The values of hydrodynamic diameters (HD) of PS1, PS2, PS3, PS4, and PS5 samples in buffers at pH 3.0, 4.0, 5.0, 6.0, and 7.0.

Type of Polymer Microparticle System	HD (nm)				
	pH 3.0	pH 4.0	pH 5.0	pH 6.0	pH 7.0
	Citric Acid/ Sodium Hydroxide/ Hydrogen Chloride	Potassium Hydrogen Phthalate	Citric Acid/ Sodium Hydroxide	Citric Acid/ Sodium Hydroxide	Potassium Dihydrogen Phosphate/ Disodium Hydrogen Phosphate
PS1	229 ± 26	526 ± 78	789 ± 42	661 ± 78	883 ± 41
PS2	280 ± 12	226 ± 15	491 ± 30	681 ± 69	1030 ± 61
PS3	271 ± 30	394 ± 35	253 ± 50	811 ± 64	819.0 ± 53
PS4	565 ± 42	387 ± 50	437 ± 42	763 ± 52	894 ± 64
PS5	599 ± 53	435 ± 72	583 ± 115	1250 ± 141	743 ± 87

**Table 3:** The characteristic thermal degradation parameters of PS1, PS2, PS3, and PS4 at 5 °C·min^−1^ in a nitrogen atmosphere at 50 mL min^−1^.

Type of Polymer Microparticle System	*T*_1_ (°C)	Rate of Mass Loss 1 (% min^−1^)	*T*_2_ (°C)	Rate of Mass Loss 2 (% min^−1^)	*T*_3_ (°C)	Rate of Mass Loss 3 (% min^−1^)	*T*_Onset_ (°C)	*T*_Endset_ (°C)	Res (%)	*T*_1.0wt%_ (°C)
PS1	50.7	0.64	293.6	2.27	357.1	1.06	185.1	396.2	22.30	33.4
PS2	54.2	0.60	295.6	2.33	392.7	1.32	180.6	403.3	29.08	33.8
PS3	56.8	0.63	296.4	2.43	393.3	1.27	180.6	404.2	30.20	33.2
PS4	55.3	0.68	297.1	2.62	381.7	0.72	176.4	405.0	23.76	32.8

**Table 4:** Intensities of selected diffraction peaks for the investigated samples; 2θ = 5–70°.

Sample	Peak 1 2θ (°)	I_1_ (Arbitrary Units)	Peak 2 2θ (°)	I_2_ (Arbitrary Units)	Peak 3 2θ (°)	I_3_ (Arbitrary Units)	Peak 4 2θ (°)	I_4_ (Arbitrary Units)
PS1	8.19	906	-	-	19.68	1627	-	-
PS2	8.21	745	-	-	19.18	1221	-	-
PS3	7.95	528	12.93	918	19.44	1281	-	-
PS4	8.02	816	12.94	1190	19.57	1617	-	-
AMPSA	-	-	11.52	22,674	15.34	21,291	23.14	17,315
KPS	-	-	-	-	-	-	27.53	34,071

## Data Availability

The primary data are available in the affiliated unit performing the assays.
